# Association between serum arginine levels and cancer risk: A community-based nested case-control study

**DOI:** 10.3389/fnut.2022.1069113

**Published:** 2022-11-17

**Authors:** Tong Liu, Xiaomeng Wang, Pingping Jia, Chenan Liu, Yaping Wei, Yun Song, Shuqun Li, Lishun Liu, Binyan Wang, Hanping Shi

**Affiliations:** ^1^Department of Gastrointestinal Surgery/Clinical Nutrition, Capital Medical University Affiliated Beijing Shijitan Hospital, Beijing, China; ^2^Beijing International Science and Technology Cooperation Base for Cancer Metabolism and Nutrition, Beijing, China; ^3^Key Laboratory of Cancer FSMP for State Market Regulation, Beijing, China; ^4^Department of Education, Capital Medical University Affiliated Beijing Shijitan Hospital, Beijing, China; ^5^Key Laboratory of Precision Nutrition and Food Quality, Ministry of Education, Department of Nutrition and Health, College of Food Sciences and Nutritional Engineering, China Agricultural University, Beijing, China; ^6^Shenzhen Evergreen Medical Institute, Shenzhen, China

**Keywords:** arginine, cancer, serum, hypertension, Chinese

## Abstract

**Objective:**

The effect of arginine on tumors appears to be bidirectional. The association of serum arginine with the risk of incident cancer remains uncovered at present. We aimed to investigate the prospective relationship of baseline serum arginine concentrations with the risk of incident cancer in hypertensive participants.

**Materials and methods:**

A nested, case-control study with 1,389 incident cancer cases and 1,389 matched controls was conducted using data from the China H-Type Hypertension Registry Study (CHHRS). Conditional logistic regression analyses were performed to evaluate the association between serum arginine and the risk of the overall, digestive system, non-digestive system, and site-specific cancer.

**Results:**

Compared with matched controls, cancer patients had higher levels of arginine (21.41 μg/mL vs. 20.88 μg/mL, *p* < 0.05). When serum arginine concentrations were assessed as quartiles, compared with participants in the lowest arginine quartile, participants in the highest arginine quartile had a 32% (OR = 1.32, 95% CI: 1.03 to 1.71), and 68% (OR = 1.68, 95% CI: 1.09 to 2.59) increased risk of overall and digestive system cancer, respectively, in the adjusted models. In the site-specific analysis, each standard deviation (SD) increment of serum arginine was independently and positively associated with the risk of colorectal cancer (OR = 1.35, 95% CI: 1.01 to 1.82) in the adjusted analysis.

**Conclusion:**

We found that hypertensive individuals with higher serum arginine levels exhibited a higher risk of overall, digestive system, and colorectal cancer.

## Introduction

As the world’s most populous country, China has made significant progress in health promotion in recent decades. However, due to increases in the severity of cancer risk factors, especially an aging population, poor diet, and higher rates of obesity, diabetes, and environmental pollution, China continues to experience a growing cancer burden with almost 22 and 27% of the global cancer cases and deaths, respectively, occurring in China in 2015 (1). The established risk factors for cancer include use of tobacco products (2), infectious agents (3), alcohol consumption (4), obesity (5), environmental pollution (6), and poor diet (7). Researchers estimate that almost 60% of cancer could be prevented by reducing these risk factors, many of which are modifiable (8). It is important to investigate numerous carcinogenic factors to determine potential screening and prevention methods.

Arginine, a semi-essential amino acid, plays a crucial role in the urea cycle and the synthesis of protein, polyamines, creatine, and nitric oxide (NO) (9). L-arginine supplementation has been demonstrated to be beneficial for endothelium-derived NO production and endothelial function in numerous studies, reducing systemic blood pressure in some forms of experimental hypertension (10). Animal studies have shown that arginine reduces white fat mass while increasing brown fat and skeletal muscle mass, increases several lipolytic enzymes, and reduces the levels of insulin resistance (IR) (11–14). Moreover, L-arginine concentrations in the intracellular environment have a direct impact on the metabolic fitness and survival capacities of T cells which are crucial for anti-tumor immunity (15). Arginine may reduce the risk of cancer due to its beneficial effect on the regulation of nutrient metabolism and T cells. In recent years, however, arginine’s role in carcinogenesis has received increasing attention because it promotes cell growth in cancerous tissues (16). Cancer microenvironments are profoundly affected by arginine availability and the activation of arginine-related pathways (16). Notably, polyamines and NO, synthesized solely from arginine, may affect tumor initiation, progression, tumor-cell adhesion, differentiation, angiogenesis, and immunosuppression (17–19). In addition, clinical trials have shown positive results with arginine deprivation in cancer therapy (20).

In short, the effect of arginine on tumors appears to be bidirectional. However, as an important amino acid, the precise role of serum arginine concentrations on the occurrence of cancer is poorly understood. This study aimed to explore the association of serum arginine levels with incident cancer risk by drawing data from a case-control study, nested within a community-based, prospective cohort among hypertensive participants, thereby providing possible implications for early diagnosis and treatment of cancer.

## Materials and methods

### Study population

The population in the current study was obtained from the China H-Type Hypertension Registry Study (CHHRS; URL^1^; unique identifier: ChiCTR1800017274), which is an ongoing, community-based, observational, and real-world registry study. The CHHRS aimed to establish a national registry of H-type hypertensive patients, to assess the prevalence, treatment, and prognosis of H-type hypertension in China. Individuals aged 18 years and over with essential hypertension, defined as seated, systolic blood pressure (SBP) ≥ 140 mmHg and/or seated, diastolic blood pressure (DBP) ≥ 90 mmHg at the screening visit were eligible for participation. Participants were excluded if they had a psychological or nervous system impairment that prevented them from giving informed consent or from being followed up according to the study protocol. There were two stages in this study: (1) recruitment and (2) observation follow-up which was scheduled every 3 months during the 3-year trial period. At each visit, SBP, DBP, heart rate, medication usage, adverse events, and study outcomes were recorded. The primary outcome was the first composite of cardiovascular events and consisted of non-fatal strokes, myocardial infarcts, and vascular deaths. Other outcomes included cancer, kidney disease, and all-cause mortality.

### Outcome assessment

Ascertainment of cancer was carried out by the Centers for Disease Control and Prevention (CDC) of Rongcheng, or through electronic linkage to hospitalizations where patients had received treatment for malignant tumors, or from active follow-up. In the absence of pathological results, potential cancer cases were further evaluated by two oncologists. Cancer cases could only be identified when the diagnoses were confirmed by both oncologists and were coded using the International Classification of Diseases, Tenth Revision (ICD-10).

### Nested case-control study

**FIGURE 1:**
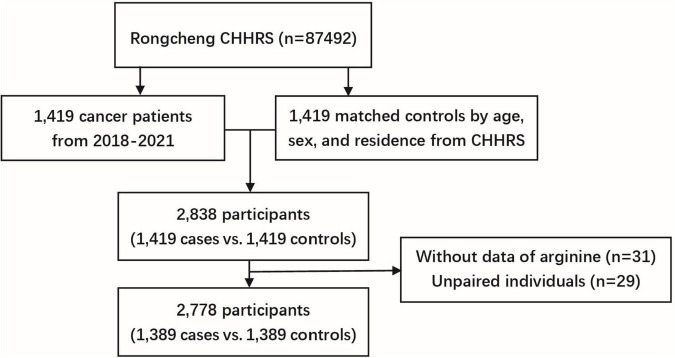
Flow chart of study participants in the nested case-control study within the HHPCP.

We conducted a case-control study nested within the CHHRS. The controls were selected from the study population who were cancer-free at the end of the follow-up period and were matched with cases by age (± 1 year), sex, and region in a 1:1 ratio. The initial sample consisted of 1,419 incident cases and 1,419 matched controls. After excluding 31 participants with missing serum arginine measurements and 29 unpaired individuals, a total of 2,778 participants (1,389 cancer cases vs. 1,389 matched controls) were included in the final analysis (Figure 1). Participants were further divided into four groups based on arginine quartiles, with cut-off values of 17.62, 21.16, and 25.64 μg/mL, respectively.

### Exposure and covariates

A morning serum sample was collected from all participants following an overnight fast at the baseline screening. Serum arginine was measured using liquid chromatography with tandem quadrupole mass spectrometry (LC-MS/MS) in a commercial lab (Beijing DIAN Medical Laboratory, China^2^). The descriptions of LC-MS/MS setting parameters, the modes and type of the instrument are described in Supplementary material. Biochemical indexes including alanine aminotransferase (ALT), albumin (ALB), triglycerides (TG), total cholesterol (TC), uric acid (UA), high-density lipoprotein cholesterol (HDL-C), fasting blood glucose (FBG), creatinine, homocysteine (HCY), and folate were analyzed using automatic clinical analyzers (Beckman Coulter) at the central laboratory of the National Clinical Research Center for Kidney Disease, (Nanfang Hospital, Guangzhou, China). Information on age, sex, marital status, education level, smoking status, alcohol consumption, sleep quality, history of chronic disease, antihypertensive drug usage, and family history of cancer was collected using a standard questionnaire. Participant height and weight were measured by trained medical staff, and body mass index (BMI) was calculated as body weight (kg) divided by the square of height (m^2^).

### Statistical analysis

Participant baseline characteristics were presented as means ± SDs, medians (IQR), and proportions for normally distributed, skewed distributed, and categorical variables; differences between the cases and controls were compared using paired *t*-tests, non-parametric Kruskal-Wallis tests, and chi-square tests (Fisher’s exact test), respectively. The dose-response relationship between arginine (per SD) and cancer risk was calculated by restricted cubic spline regression (RCS). Odds ratios (ORs) and 95% confidence intervals (CIs) for the association of serum arginine levels (per SD, and quartiles) with overall, digestive system, and non-digestive system cancer risk were estimated using conditional logistic regression with models unadjusted and adjusted for the variables including BMI, smoking status, alcohol drinking status, SBP, TG, TC, UA, glucose, HDL-C, creatinine, ALB, ALT, HCY, sleep quality, anti-hypertensive drug usage, and family history of cancer. In addition, we further explored the effect of serum arginine levels on the occurrence of site-specific cancers. A subgroup analysis on the association was also conducted on the variables age (median, < 69 vs. ≥ 69 years), sex, smoking status (never vs. past or current), drinking status (never vs. past or current), folate levels (median, < 6.13 vs. ≥ 6.13 ng/mL), and BMI (< 24 vs. 24–27.9 vs. ≥ 28 kg/m^2^). The association of serum arginine with cancer risk was reanalyzed after further dividing participants by their median follow-up interval according to the time of cancer occurrence (< median vs. ≥ median follow-up period) to avoid any possible influence of preclinical disease on the results. A two-tailed *P* < 0.05 was considered statistically significant in all analyses. R software (version 3.4.1^3^) and SAS (version 9.4) were used for all statistical analyses.

## Results

### Baseline characteristics of the participants

The current study included 1,389 cancer cases, with 543 digestive system cancer cases and 846 non-digestive system cancer cases. The most common cancer types were lung (*n* = 361), followed by colorectal (*n* = 180), gastric (*n* = 160), liver (*n* = 107), breast (*n* = 85), head and neck (*n* = 82), prostatic (*n* = 58), lymphoma and leukemia (*n* = 57), pancreatic (*n* = 48), uterine and cervical (*n* = 47), and bladder cancer (*n* = 41). Table 1 shows the characteristics of cancer patients and matched controls. Compared with matched controls, cancer patients had higher levels of arginine (21.41 μg/mL vs. 20.88 μg/mL, *p* < 0.05). Significant differences were found in concentrations of ALB, and percentage rates of the current smoker, history of stroke, history of CHD, poor sleep quality, and antihypertensive drug usage between cases and matched controls. In addition, there were no differences between cancer patients and controls in terms of age, sex, BMI, blood pressure, ALT, TG, TC, UA, HDL-C, FBG, creatinine, folate, HCY, marital status, current drinker, history of CKD, and family history of cancer (all *p*-values for differences > 0.05).

**TABLE 1:** Baseline characteristics of the cancer cases and matched controls.

Variables	Controls (*n* = 1,389)	Cases (*n* = 1,389)	*p*-value
Age, y	69.32 ± 7.76	69.32 ± 7.76	0.999
Males, n (%)	779 (56.08)	779 (56.08)	1.000
BMI, kg/m^2^	25.73 ± 3.60	25.72 ± 3.83	0.953
Baseline SBP, mmHg	148.48 ± 21.26	147.52 ± 21.30	0.237
Baseline DBP, mmHg	83.73 ± 11.33	83.07 ± 11.76	0.134
ALT, U/L	8.05 (7.0, 13.0)	10.0 (7.0, 14.0)	0.148
ALB, g/L	45.43 ± 2.45	44.75 ± 2.97	< 0.001
TG, mmol/L	1.21 (0.86, 1.77)	1.19 (0.84, 1.80)	0.630
TC, mmol/L	6.51 ± 1.23	6.44 ± 1.30	0.202
UA, μmol/L	320.0 (269.0, 371.0)	314.0 (264.0, 374.0)	0.393
HDL-C, mmol/L	1.23 ± 0.24	1.22 ± 0.27	0.795
FBG, mmol/L	6.25 ± 1.71	6.29 ± 1.83	0.626
Creatinine, μmol/L	45.84 (27.03, 51.00)	52.0 (10.0, 64.0)	0.748
Folate, ng/mL	6.14 (4.03,9.66)	6.11 (4.22, 10.12)	0.466
HCY, μmol/L	12.01 (10.31, 14.66)	12.25 (10.06, 15.03)	0.886
Arginine, μg/mL	20.88 (17.34, 25.11)	21.41 (17.94, 26.25)	0.001
Marital status, [married, n (%)]	1144 (82.36)	1178 (84.81)	0.354
High school education or above, n (%)	108 (7.78)	103 (7.42)	0.720
Current smoker, n (%)	334 (24.05)	401 (28.87)	0.011
Current drinker, n (%)	388 (27.93)	370 (26.64)	0.359
History of CKD, n (%)	14 (1.01)	25 (1.80)	0.076
History of CHD, n (%)	0 (0)	165 (11.88)	< 0.001*
History of stroke, n (%)	0 (0)	64 (4.61)	< 0.001*
Family history of cancer, n (%)	47 (3.38)	50 (3.60)	0.918
Poor sleep quality, n (%)	191 (13.75)	259 (18.65)	0.002
Antihypertensive drug usage, n (%)	494 (35.57)	557 (40.10)	0.014

BMI, body mass index; SBP, systolic blood pressure; DBP, diastolic blood pressure; ALT, alanine aminotransferase; ALB, albumin; TG, triglycerides; TC, total cholesterol; UA, uric acid; HDL-C, high-density lipoprotein cholesterol; FBG, fasting blood glucose; HCY, homocysteine; CKD, chronic kidney disease; CHD, coronary heart disease. *Compared by Fisher’s exact test.

### Association of arginine with the risk of cancer

**FIGURE 2:**
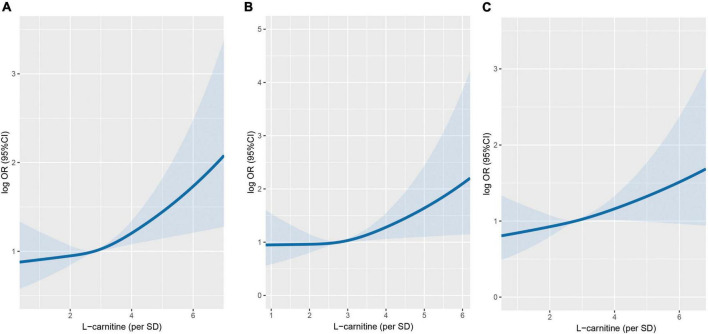
Association between serum arginine concentrations and cancer risk using restricted cubic spline (RCS). **(A)** Overall cancer. **(B)** Digestive system cancer. **(C)** Non-digestive system cancer. Models were adjusted for BMI, smoking status, alcohol drinking, systolic blood pressure, triglycerides, cholesterol, uric acid, glucose, HDL-C, creatinine, ALB, ALT, HCY, sleep quality, antihypertensive drug usage, and family history of cancer.

Figure 2 shows the dose-response relationship between arginine concentrations (per SD) and incident cancer risk. Arginine was found to be positively, and non-linearly correlated with the risk of overall and digestive system cancer, but not with the risk of non-digestive system cancer. Tables 2, 3 show the ORs (95% CI) of arginine associated with the risk of overall, digestive system cancer, and non-digestive system cancer. Each standard deviation (SD) increment of serum arginine concentration significantly elevated the risk of overall cancer (OR = 1.13, 95% CI: 1.03 to 1.24) and digestive system cancer (OR = 1.21, 95% CI: 1.04 to 1.42) in the multivariate analysis. Compared with participants in the lowest arginine quartile (Q1), patients in the highest arginine quartile (Q4) had a 32% (OR = 1.32, 95% CI: 1.03 to 1.71), and 68% (OR = 1.68, 95% CI: 1.09 to 2.59) increased risk of overall and digestive system cancer in the adjusted models, respectively. Table 4 shows the effect of arginine on the occurrence of site-specific cancers. Each standard deviation (SD) increment of serum arginine was independently and positively associated with the risk of colorectal cancer in the adjusted analysis (OR = 1.35, 95% CI: 1.01 to 1.82).

**TABLE 2:** The association of serum arginine with overall cancer risk.

Arginine (μg/mL)	Cases/Controls (Ratio 1:1)	Crude model		Adjusted model	
			
		OR (95% CI)	*p*-value	OR (95% CI)	*p*-value
**Per SD**	1389/1389	**1.16 (1.07, 1.26)**	**< 0.001**	**1.13 (1.03, 1.24)**	**0.007**
**Quartiles**					
Q1 (< 17.62)	327/368	Ref.		Ref.	
Q2 (17.62– < 21.16)	343/352	1.12 (0.90, 1.38)	0.309	1.05 (0.83, 1.32)	0.681
Q3 (21.16– < 25.64)	339/354	1.11 (0.89, 1.38)	0.348	1.13 (0.89, 1.45)	0.321
Q4 (≥ 25.64)	380/315	**1.43 (1.14, 1.79)**	**0.002**	**1.32 (1.03, 1.71)**	**0.031**

Models were adjusted for ALT, ALB, BMI, smoking status, alcohol drinking, SBP, TC, TG, UA, HDL-C, glucose, creatinine, folate, HCY, sleep quality, antihypertensive medication, and family history of cancer. Statistically significant values are shown in bold with all *p* values < 0.05.

**TABLE 3:** The association of arginine with the digestive system and non-digestive system cancer risk.

Arginine (μg/mL)	Digestive system			Non-digestive system		
		
	Cases/Controls	OR (95% CI)	*p*-value	Cases/Controls	OR (95% CI)	*p*-value
**Per SD**	543/543	**1.21 (1.04, 1.42)**	**0.017**	846/846	1.09 (0.97, 1.22)	0.135
**Quartiles**						
Q1 (< 17.62)	133/153	Ref.		194/215	Ref.	
Q2 (17.62– < 21.16)	129/134	1.08 (0.73, 1.59)	0.694	214/218	1.01 (075, 1.36)	0.953
Q3 (21.16– < 25.64)	139/139	1.30 (0.86, 1.96)	0.207	200/215	1.06 (0.78, 1.46)	0.704
Q4 (≥ 25.64)	142/117	**1.68 (1.09, 2.59)**	**0.018**	238/198	1.15 (0.83, 1.60)	0.397

Models were adjusted for ALT, ALB, BMI, smoking status, alcohol drinking, SBP, TC, TG, UA, HDL-C, glucose, creatinine, folate, HCY, sleep quality, antihypertensive medication usage, and family history of cancer. Statistically significant values are shown in bold with all *p* values < 0.05.

**TABLE 4:** The association of serum arginine (per SD) with site-specific cancer risk.

Arginine (μg/mL)	Cases/Control (Ratio 1:1)	Crude model		Adjusted model	
			
		OR (95% CI)	*p*-value	OR (95% CI)	*p*-value
Lung cancer	361/361	**1.22 (1.04, 1.43)**	**0.015**	1.15 (0.96, 1.37)	0.131
Colorectal cancer	180/180	**1.40 (1.10, 1.76)**	**0.006**	**1.35 (1.01, 1.82)**	**0.048**
Gastric cancer	160/160	1.23 (0.95, 1.59)	0.118	1.34 (0.97, 1.86)	0.077
Liver cancer	107/107	0.87 (0.63, 1.19)	0.379	0.89 (0.58, 1.36)	0.589
Breast cancer	85/85	1.13 (0.82, 1.51)	0.487	1.05 (0.70, 1.59)	0.813
Head and neck cancer	82/82	**1.67 (1.14, 2.44)**	**0.008**	1.48 (0.93, 2.35)	0.096
Lymphoma and leukemia	57/57	1.19 (0.80, 1.78)	0.167	2.23 (0.72, 6.93)	0.167
Prostatic cancer	58/58	0.97 (0.68, 1.38)	0.878	0.90 (0.53, 1.53)	0.695
Pancreatic cancer	48/48	1.06 (0.68, 1.68)	0.788	0.57 (0.16.2.12)	0.405
Bladder cancer	41/41	1.07 (0.71, 1.59)	0.759	0.46 (0.14, 1.55)	0.210
Uterine and cervical cancer	47/47	0.90 (0.55, 1.47)	0.664	0.66 (0.31, 1.40)	0.282

Models were adjusted for ALT, ALB, BMI, smoking status, alcohol drinking, SBP, TC, TG, UA, HDL-C, glucose, creatinine, HCY, sleep quality, antihypertensive medication, and family history of cancer. Statistically significant values are shown in bold with all *p* values < 0.05.

### Subgroup analyses

**FIGURE 3:**
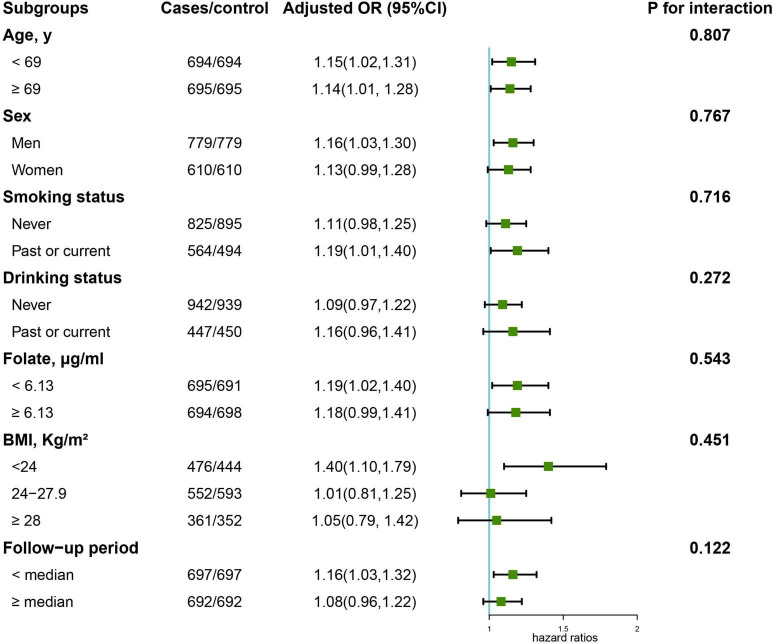
Stratified analyses of the association of serum arginine (per SD) with the risk of overall cancer. Models were adjusted for BMI, smoking status, alcohol drinking, systolic blood pressure, triglycerides, cholesterol, uric acid, glucose, HDL-C, creatinine, ALB, ALT, HCY, sleep quality, antihypertensive drug usage, and family history of cancer except for the stratified factors.

Figure 3 illustrates the results of the subgroup analysis of the association between serum arginine concentrations and overall cancer risk. None of the factors, including age, sex, smoking status, drinking status, folate levels, body mass index, and follow-up period, had an effect on the association between arginine concentrations and overall cancer risk (all *p* for interaction < 0.05). Significant, positive associations of arginine levels with overall cancer risk were found among all age subgroups, males, past/current smokers, and those with lower folic acid levels, normal BMI and whose cancer occurred prior to the median of the follow-up period.

## Discussion

In this case-control study nested within a population-based, prospective cohort study, we found that hypertensive individuals with higher serum arginine levels exhibited a higher risk of overall cancer. Significant associations were similarly observed for digestive system cancer, especially colorectal cancer. We also observed significant associations of arginine levels with overall cancer risk among all age subgroups, males, past/current smokers, and individuals with lower folic acid levels, normal BMI and with cancer occurring before the median follow-up period.

This study is the first to find that serum arginine levels are positively associated with overall and colorectal cancer risk in hypertensive cancer-free participants. However, results from several previous studies partly support our findings as follows: A study including *in vivo* results in mice and epidemiologic results in human cancer cases found that an arginine diet resulted in higher tumor grades in mice, and meat consumption (a major source of dietary arginine) resulted in adverse outcomes for patients suffering from familial CRC (21); Yerushalmi HF et al. evaluated the roles of dietary arginine and inducible nitric oxide synthase (NOS2) in Apc-dependent intestinal tumorigenesis in Min mice with or without a functional NOS2 gene, and found that dietary arginine increased colon tumorigenesis in ApcMin/ + mice (22); Some tumors (auxotrophic tumors) require arginine for growth, and disturbances in arginine metabolism is a distinct feature of the presence of a malignant tumor. Several previous studies also found a significant decrease in arginine levels among cancer patients (23–25).

Nitric Oxide (NO) is a ubiquitous signal transduction molecule generated by arginine metabolism, and NO has been linked to a large number of cancer-related events (16). Despite its simplistic biochemistry, NO plays an extremely complex role in tumor biology. One study found that ulcerative colitis patients might be more likely to develop cancer because chronic colonic inflammation increases NO production (17). Furthermore, NO has also been implicated in the development of cholangiocarcinoma (18) and hepatocellular carcinoma (26) in experimental studies. Moreover, several lines of investigation have suggested that arginine-derived NO could influence the initiation, progression, apoptosis, angiogenesis, and metastasis of numerous neoplasms (27–29).

Many cancer cells show deficiencies in arginine metabolic pathways and thus rely on the uptake of arginine for rapid metabolism and proliferation. The auxotrophy of cancer cells to arginine renders these cancer cells vulnerable to the deprivation of this specific amino acid. Thus, arginine deprivation has become an accessible choice for cancer treatment. Arginine deprivation drugs are necessary since dietary restriction only reduces circulating arginine by 30% (30). Thus far, two types of protein drugs, arginine deiminase (ADI) and human arginase (hArg), have been developed to deplete arginine for cancer treatment (31, 32).

It is evident that arginine, and its availability, affect lymphocyte performance. Infiltrating macrophages (TIM) possess high arginase levels, which may modulate arginine availability in the tumor’s microenvironment. This inhibiting effect of arginine supplementation on immunogenic tumors may be due to its beneficial effects on the immune system, particularly macrophages, natural killer cells, and T cell cytotoxicity (33). Early studies have found that arginine supplementation increases T-cell proliferation and reverses post-traumatic T-cell suppression (34, 35). Arginine is also shown to enhance T-cell responses in nude mice (36), although those effects have not been observed in other immune-activated states (37). A nested, case-control study within the European Prospective Investigation into Cancer cohort (EPIC) including 1,124 breast cancer cases and 1,124 matched controls, found that concentrations of arginine were inversely associated with breast cancer risk (OR [per SD] = 0.79, 95% CI = 0.70–0.90) (38).

Recently, other amino acids rather than arginine are demonstrated to be closely associated with cancer development. First, asparagine (Asn) suppresses apoptosis by negatively modulating endoplasmic reticulum stress and translation-dependent apoptosis (39). Cancer cells that express a low level or are deficient in Asn synthetase (ASNS) may be induced by Asn starvation (40). Second, a high level of glutamine (Gln) is essential for maintaining TCA cycle anaplerosis and supporting the survival of cancer cells (41). Third, cancer cells exhibit elevated levels of ROS intracellularly due to alterations in the microenvironment and metabolism (42). As a counterbalance to excessive ROS levels, tumor cells maintain reduced forms of glutathione (GSH) in part to produce more reducing equivalents (43). As cysteine (Cys) is one of the building blocks of GSH, elevated production of Cys may exhaust endogenous sources of the substance (44).

The main strength of the current study is its novelty, for it uncovers the association of serum arginine levels with the risk of incident cancer among a hypertensive Chinese population. Furthermore, it has the advantage of being a nested, case-control study that was derived from a large, prospective cohort study, thus avoiding recall bias. The serum arginine levels of participants were determined before any cancer diagnosis, eliminating the possibility of a causal association.

Several limitations should also be noted in the current study. First, serum arginine levels were only measured at baseline, regular measurements would have provided a better understanding of the dynamic relationship between cancer risk and changes in arginine levels. Second, the small number of cancer cases and the short follow-up period prevented further analysis on subtypes of cancer, a larger population is needed to validate the findings. Third, this case-control study was nested within CHHRS, which was designed to assess the prevalence, treatment, and prognosis of H-type hypertension in China. Therefore, the population in this study was hypertensive adults. It is unclear if the findings can be generalized to non-hypertensive populations. However, blood pressure was also adjusted for in the multivariate analysis, which minimized the impact of blood pressure in the current study. Fourth, although we found a positive association between arginine and cancer risk, whether the higher levels of blood arginine are the possible cause or as the major precursor for synthesis of cancer-associated compounds such as NO, and NO synthetase need to be better elucidated in the future studies. Last, our results were based on a nested, case-control study; further explorations of this association in large-scale cohort studies and randomized trials are needed.

## Conclusion

In this nested, case-control study among a hypertensive adult population, we found an independent effect of serum arginine concentrations on the risk of incident cancer. In light of the heavy burden and the fatality of cancer in China and throughout the world, our study’s findings could provide a safe and straightforward mechanism for cancer prevention.

## Data availability statement

The raw data supporting the conclusions of this article will be made available by the authors, without undue reservation.

## Ethics statement

The studies involving human participants were reviewed and approved by the CHHRS and the present study were approved by the Ethics Committee of the Institute of Biomedicine, Anhui Medical University, Hefei, China. The patients/participants provided their written informed consent to participate in this study.

## Author contributions

TL: methodology, software, and writing—original draft preparation. XW: writing—reviewing and editing. PJ: methodology and software. CL: visualization. YW, LL, and BW: supervision and validation. YS: investigation. SL: supervision. HS: conceptualization, funding acquisition, resources, and supervision. All authors contributed to the article and approved the submitted version.

## Footnotes

1
http://www.chictr.org.cn/showprojen.aspx?proj=28262

2
http://www.dazd.cn

3
http://www.R-project.org
