# Supplementary material for: Association between serum arginine levels and cancer risk: A community-based nested case-control study
Source: Front Nutr. 2022 Nov 17;9:1069113. doi: 10.3389/fnut.2022.1069113 (PMC9712959; doi:10.3389/fnut.2022.1069113)
Supplement: Supplementary file 1 [file Data_Sheet_1.docx]

### Chromatographic mass spectrometry conditions

1. **Chromatographic conditions**
   1. **Column:** Water ACQUITY UPLC® BEH HILIC (2.1x100mm, 1.7µm) (or equivalent column)
   2. **Column temperature:** 40℃
   3. **Autosampler temperature:** 4℃
   4. **Flow rate:** 0.8 ml/min
   5. **Injection volume:** 10 μl
   6. **method gradient**

Table S1. Elution gradient

| time (min) | Flow rate (mL/min) | A (%) | B (%) |
| --- | --- | --- | --- |
| 0 | 0.8 | 10 | 90 |
| 1.00 | 0.8 | 10 | 90 |
| 1.01 | 0.8 | 15 | 85 |
| 2.30 | 0.8 | 15 | 85 |
| 2.31 | 0.8 | 10 | 90 |

- - - 1. **Mobile phase:**

A: Water (containing 10 mmol/L ammonium acetate, 0.5 % formic acid)

B: 95% acetonitrile-water (containing 10 mmol/L ammonium acetate, 0.5 % formic acid)

1. **MS conditions**
   1. **Common mass spectrometry parameters**

Table S2. Mass spectrometry parameter information

| source of ion | Electrospray ionization source |
| --- | --- |
| ion mode | positive mode |
| Collision Gas (CAD) | 6 |
| Curtain Air (CUR) | 26 |
| Atomizing current (NC) | 3.0 |
| Atomizing gas (Gas1) | 45 |
| Auxiliary gas (Gas2) | 45 |
| Spray current (IS) | 5500 |
| Atomization temperature (TEM) | 650 |
| scan mode | Multiple Reaction Monitoring (MRM) |

- 1. **Transition parameters of each analyte in multiple reaction monitoring (MRM)**

Table S3. Transition parameter information

| ID | Q1Mass | Q3Mass | Dwell Time (ms) | DP (volts) | CE (volts) | EP(V) | CXP(V) |
| --- | --- | --- | --- | --- | --- | --- | --- |
| Arginine | 175.1 | 70.2 | 20.0 | 60 | 28 | 10 | 6 |
| Arginine-^13^C_6_ | 181.2 | 74.0 _ | 20.0 | 80 | 30 | 10 | 6 |

- 1. **Other parameter information**

1. **Scheduled MRM:** Check Basic;
2. **Period Summary:** Duration: 4.0(min);
3. **Delay Time:** 0 (sec);
4. **Cycles:** 400, Cycle: 0.6002(sec)
5. **Target for s MRM:** Target Scan Time (per s MRM expt ): 0.3 ( sec)
6. **MRM detection window:** 20.0 (sec)
